# Synthesis and Bioevaluation of Iodine-131 Directly Labeled Cyclic RGD-PEGylated Gold Nanorods for Tumor-Targeted Imaging

**DOI:** 10.1155/2017/6081724

**Published:** 2017-12-24

**Authors:** Yingying Zhang, Yongxue Zhang, Lianglan Yin, Xiaotian Xia, Fan Hu, QingYao Liu, Chunxia Qin, Xiaoli Lan

**Affiliations:** ^1^Department of Nuclear Medicine, Union Hospital, Tongji Medical College, Huazhong University of Science and Technology, Wuhan 430022, China; ^2^Hubei Key Laboratory of Molecular Imaging, Union Hospital, Tongji Medical College, Huazhong University of Science and Technology, Wuhan 430022, China

## Abstract

**Introduction:**

Radiolabeled gold nanoparticles play an important role in biomedical application. The aim of this study was to prepare iodine-131 (^131^I)-labeled gold nanorods (GNRs) conjugated with cyclic RGD and evaluate its biological characteristics for targeted imaging of integrin *α*_v_*β*_3_-expressing tumors.

**Methods:**

HS-PEG_(5000)_-COOH molecules were applied to replace CTAB covering the surface of bare GNRs for better biocompatibility, and c(RGDfK) peptides were conjugated onto the carboxyl terminal of GNR-PEG-COOH via EDC/NHS coupling reactions. The nanoconjugate was characterized, and ^131^I was directly tagged on the surface of GNRs via AuI bonds for SPECT/CT imaging. We preliminarily studied the characteristics of the probe and its feasibility for tumor-targeting SPECT/CT imaging.

**Results:**

The [^131^I]GNR-PEG-cRGD probe was prepared in a simple and rapid manner and was stable in both PBS and fetal bovine serum. It targeted selectively and could be taken up by tumor cells mainly via integrin *α*_v_*β*_3_-receptor-mediated endocytosis. In vivo imaging, biodistribution, and autoradiography results showed evident tumor uptake in integrin *α*_v_*β*_3_-expressing tumors.

**Conclusions:**

These promising results showed that this smart nanoprobe can be used for angiogenesis-targeted SPECT/CT imaging. Furthermore, the nanoprobe possesses a remarkable capacity for highly efficient photothermal conversion in the near-infrared region, suggesting its potential as a multifunctional theranostic agent.

## 1. Introduction

Ideal physicochemical properties, a high binding affinity for selected molecules with thiol terminal groups, and remarkable photoacoustic features provide gold nanoparticles (GNPs) with significant capabilities for biomedical applications [1, 2]. Different forms of gold nanostructures, such as GNPs [3], gold nanorods (GNRs) [4], gold nanocages [5], gold nanospheres [6], and gold nanoshells [7], have been investigated for molecular imaging and therapy. As a representative GNP, GNRs have attracted considerable attention in recent years because of their small size, ease of preparation and bioconjugation, strong absorption and scattering properties, and well-characterized biocompatibility [8]. The long surface plasmon resonance (LSPR) of GNRs can be finely tuned by the aspect ratio [9], which gives rise to many exciting possibilities for biosensing, optical imaging, photothermal therapy, and drug delivery. With a proper aspect ratio, the LSPR of GNRs can be located in the near-infrared region (NIR; 650–900 nm) that is particularly suitable for in vivo imaging and photothermal therapy [9–11].

The high reaction activity of the surface in the crystal structure of GNRs allows multiple functionalizations including target ligands (e.g., peptides [12], folic acid [13], and antibodies [14]) and imaging agents (e.g., fluorescent, radionuclide, and contrast reagents) [15, 16]. Cetyltrimethylammonium bromide (CTAB), a kind of cationic surfactant stabilizer, is essential for the synthesis of GNRs. However, CTAB molecules exhibit strong cytotoxicity that can induce cell apoptosis and autophagy by damaging mitochondria and generating intracellular reactive oxygen species [17]. Fortunately, CTAB can be replaced or conjugated with many functional groups [2]. Introducing polyethylene glycol (PEG) to the surface of nanoparticles achieves better biocompatibility and lower cytotoxicity by decreasing the opsonization effect and minimizing nonspecific uptake by the reticuloendothelial system in vivo for a longer blood circulation time [8]. In addition, it has been shown that halide ions chemisorb onto the gold surface with Au-X, and its binding strength varies as I > Br > Cl [18]. Iodine-131 (^131^I; *t*_1/2_ = 8.01 days), a radionuclide with gamma emission of 364 keV and beta emission of 0.608 keV, provides imaging feasibility and a beta-emitting therapeutic effect, which makes it the optimal choice for application as a theranostic agent [19].

Extensive angiogenesis exists in solid tumors, which can be used as a diagnostic and therapeutic target. Integrin *α*_v_*β*_3_ is a cell adhesion molecule overexpressed on most tumor cells for regulation of angiogenesis and plays important roles in various stages, such as malignant transformation, tumor growth, progression, invasion, and metastasis [20]. An Arg-Gly-Asp- (RGD-) based strategy to target integrin *α*_v_*β*_3_ is one of the most promising and best studied in oncological research [20], especially cyclic RGD (cRGD) peptides, which have higher affinity, selectivity, and stability than linear peptides [16]. Furthermore, hypervasculature, a defective vascular architecture, poor lymphatic drainage, or recovery system, and greatly increased production of a number of permeability mediators facilitate nanosized particle extravasation from the blood pool, which can be retained in solid tumors, known as the enhanced permeability and retention (EPR) effect [21]. Therefore, cRGD-conjugated nanodrugs can accumulate in tumor tissues actively through target molecules and passively because of the EPR effect, resulting in increased curative efficacy and reduced side effects [22, 23].

Based on the above theoretical fundamentals, we synthesized a smart multifunctional nanoprobe, ^131^I-labeled, cRGD-conjugated PEG-modified GNRs and evaluated the feasibility of the nanoprobe for tumor-targeted imaging by in vitro cell experiments and in vivo tumor-bearing mouse imaging.

## 2. Materials and Methods

### 2.1. Chemicals and Materials

The chemicals and materials included a gold colloid solution (GNR-PEG) (Xi'an Ruixi Biological Tech. Co., Ltd., Xi'an, China), N-(3-dimethylaminopropyl)-N′-ethylcarbodiimide hydrochloride (EDC·HCl) (Aladdin Bio-Chem Tech. Co., Ltd, Shanghai, China), N-hydroxysuccinimide (NHS) (Sinopharm Chemical Reagent Co., Ltd., Shanghai, China), cyclo (Arg-Gly-Asp-d-Phe-Lys) [c(RGDfK)] (GL Biochem Ltd., Shanghai, China), sodium iodide-131 ([^131^I]NaI) (Atom High Tech., Beijing, China), and instant thin-layer chromatography-silica gel (iTLC-SG) (Agilent Tech, Santa Clara, CA, USA).

### 2.2. Synthesis of [^131^I]GNR-PEG-cRGD

**Figure 1:**
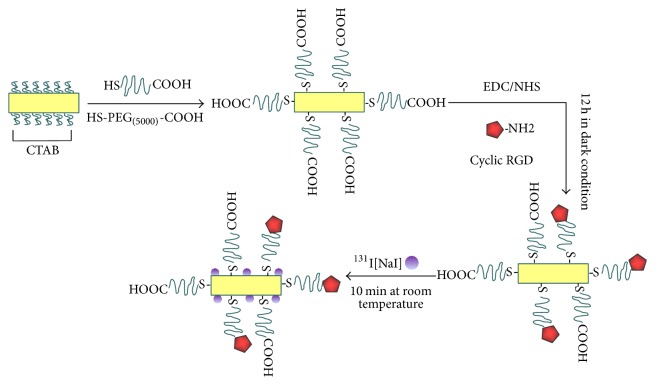
Scheme of the [^131^I]GNR-PEG-cRGD preparation procedure.

A scheme of the [^131^I]GNR-PEG-cRGD preparation procedure is shown in Figure 1.

First, EDC·HCl (5.88 × 10^-2 ^mmol) and NHS (5.88 × 10^-2 ^mmol) were individually dissolved in 500 *μ*L ultrapure deionized (DI) water, added to 1 mL of a GNR-PEG solution (0.59 mg Au/mL), and allowed to react overnight in the dark at room temperature. Subsequently, 500 *μ*L c(RGDfK) peptide (5.89 × 10^-3 ^mmol) was added to the reaction mixture, followed by stirring for 12 h in the dark at room temperature. Sonication was conducted discontinuously during the reaction process to avoid formation of precipitates or aggregates. The final product was purified by centrifugation (8000 rpm for 15 min at 4°C), redispersed in 1 mL DI water, and stored at 4°C in the dark.

Radiolabeling was performed before use. Briefly, [^131^I]NaI (1111 MBq/mL) was added to the GNR-PEG-cRGD solution (118 *μ*g/mL) and allowed to react for 15 min at room temperature, followed by centrifugation (8000 rpm for 15 min at 4°C), and then redispersed in 1 mL phosphate-buffered saline (PBS).

### 2.3. Characterization of GNRs

The morphology and size of GNRs were characterized by transmission electron microscopy (TEM). Optical absorption spectra were measured on a UV-Vis-NIR spectrophotometer (722S, Jinghua Instrument, Shanghai, China). The hydrodynamic diameter and zeta potential were measured by ZetaPALS zeta potential analyzer (Brookhaven Instrument Corp., Holtsville, NY, USA). The in vitro stability of [^131^I]GNR-PEG-cRGD in PBS and fetal bovine serum (FBS) was determined by mixing 0.1 mL [^131^I]GNR-PEG-cRGD with an equal volume of PBS/FBS and incubating at 37°C for 48 h. Radiochemical stability was monitored by iTLC-SG with a 0.9% sodium chloride solution as the solvent on a radioactive chromatography scanner (Zhongcheng, Hefei, China) at 6, 12, 24, and 48 h.

### 2.4. Cell Culture and Analysis of Integrin *α*_v_*β*_3_ Expression

Integrin *α*_v_*β*_3_-positive B16F10 mouse malignant melanoma cells and integrin *α*_v_*β*_3_-negative MCF-7 human breast cancer cells (American Type Culture Collection, Manassas, VA, USA) were cultured in Dulbecco's modified Eagle's medium (Gibco, Carlsbad, CA, USA) containing 10% (v/v) FBS (Gibco) and 1% antibiotics (100 U/ml penicillin and 100 U/ml streptomycin; Beyotime, Shanghai, China) at 37°C with 5% CO_2_. The expression of integrin *α*_v_*β*_3_ was confirmed by immunofluorescence with a primary anti-integrin *α*_v_*β*_3_ antibody (1 : 100, Bioss, Beijing, China) and Cy3-conjugated goat anti-rabbit secondary antibody (1 : 50, Aspen, Wuhan, China) as described previously [24]. Anti-rabbit IgG (Cell Signaling Technology, Inc., USA) instead of the primary antibody was used as the control.

### 2.5. In Vitro Cell Binding Assay

B16F10 and MCF-7 cells were seeded in 24-well plates at a density of 2 × 10^5^ cells per well, incubated at 37°C overnight, and then treated with 0.8 mL [^131^I]GNR-PEG-cRGD (0.074 MBq/well) at 37°C for 30, 60, 120, and 240 min. The medium was then removed, and the cells were collected and washed twice with PBS. The cell pellet was lysed with 1 N NaOH and then washed twice with PBS. Radioactivity was measured with a WIZARD *γ*-counter (PerkinElmer, MA, USA). The percentage of cellular uptake activity was calculated. Nonspecific binding was tested in parallel using B16F10 cells preincubated with excess free c(RGDfK) peptide for 1 h.

### 2.6. Animal Model, In Vivo Imaging, and Biodistribution Analyses

All animal experiments were performed in compliance with the Institutional Animal Care and Use Committee of Huazhong University of Science and Technology. MCF-7 cells (7 × 10^6^) suspended in 100 *μ*L PBS were subcutaneously injected into the right shoulder flank of BALB/C-nu/nu mice (female, 3-4 weeks old, Beijing HFK Bioscience Co., Led, China), and 2 × 10^5^ B16F10 cells were implanted in C57BL/6 mice (female, 5-6 weeks, Wuhan Centers for Disease Prevention & Control, China).

When the tumor diameter reached about 10 mm, [^131^I]GNR-PEG-cRGD (5.55–7.4 MBq, 100 *μ*L) was injected via the tail vein. Static images were acquired using a Symbia T6 SPECT/CT scanner (Siemens, Germany) under 2% pentobarbital sodium (Boster, Wuhan, China) anesthesia at 1, 3, 6, 9, and 12 h after injection. For blocking experiments, 6 mg c(RGDfK) was administrated at 1 h before injection of [^131^I]GNR-PEG-cRGD into B16F10 tumor-bearing mice.

For biodistribution analysis, 1.48–1.85 MBq [^131^I]GNR-PEG-cRGD was intravenously injected into tumor-bearing mice, and then tissue dissection was carried out at 1, 3, and 6 h after injection. Tumors and organs of interest (blood, brain, heart, lung, liver, spleen, kidney, stomach, intestine, muscle, bone, and thyroid) were collected, weighed, and analyzed using the *γ*-counter. Tissue radioactivity was expressed as the percentage of injected dose per gram of tissue (% ID/g).

### 2.7. Autoradiography and Immunofluorescence

Tumors, muscles, lungs, livers, spleens, and kidneys were excised and fixed in 4% paraformaldehyde. Frozen sections (20 *μ*m thick) were prepared and placed on a phosphor screen for 40 min and then analyzed on a Cyclone Plus Phosphor Scanning System (PerkinElmer, USA). Regions of interest (ROIs) were drawn to quantify the radioactivity.

Immunofluorescence staining of tumors was performed as described above.

### 2.8. Statistical Analysis

Data are shown as the mean ± standard deviation. Comparisons between groups were made using the Student's *t*-test. *p* < 0.05 was considered to be statistically significant.

## 3. Results

### 3.1. Synthesis of [^131^I]GNR-PEG-cRGD

**Figure 2:**
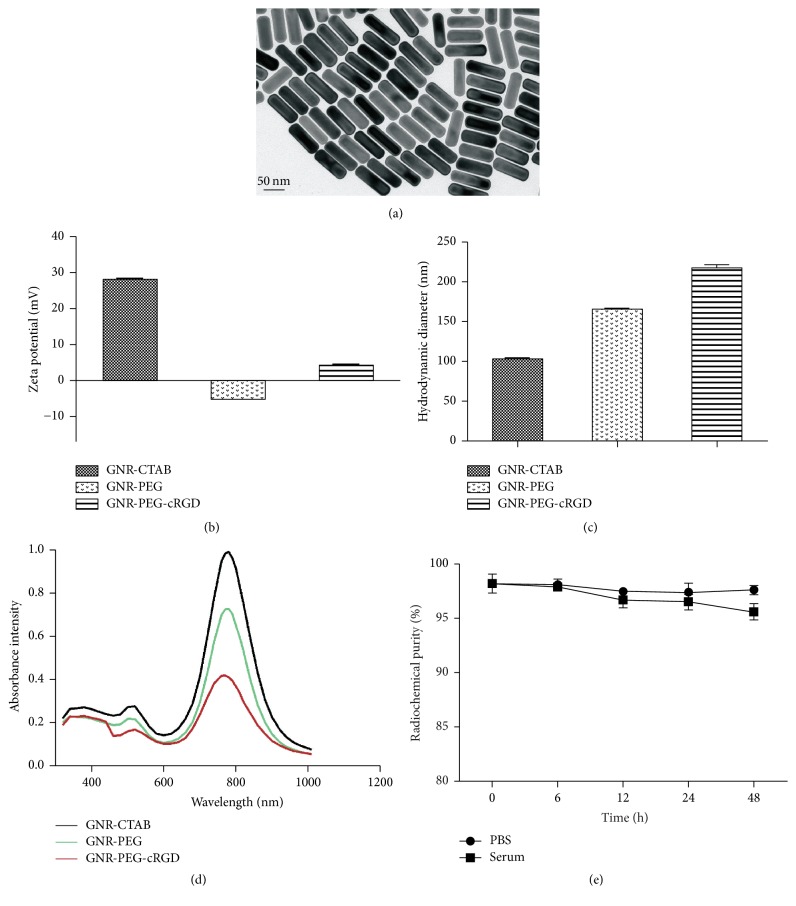
Characterization of GNR-PEG-cRGD. (a) TEM of GNR-PEG. Zeta potentials (b), hydrodynamic diameter (c), and UV-vis spectra (d) of GNR-CTAB, GNR-PEG, and GNR-PEG-cRGD. (e) In vitro stability of [^131^I]GNR-PEG-cRGD in PBS and FBS at 6, 12, 24, and 48 h after labeling.

TEM images showed that PEGylated GNRs were well dispersed with a narrow size distribution and exhibited a rod shape with an average aspect ratio of 3.8 (93.4 nm in length and 24.8 nm in width) (Figure 2(a)). The successful modification of GNRs with PEG and conjugation of c(RGDfK) peptides were confirmed by zeta potentials (Figure 2(b)) and the hydrodynamic diameter (Figure 2(c)). Cetyltrimethylammonium bromide (CTAB) is a cationic surfactant, so the zeta potential of GNR coated with CTAB was 28.13 ± 0.59 mV. When CTAB was replaced with a long chain structure HS-PEG-COOH molecule, the zeta potential of GNR-PEG shifted in negative direction to −5.17 ± 0.60 mV due to the carboxyl end group of HS-PEG-COOH molecule dissociated in aqueous solution and showed negative potential. On conjugation with c(RGDfK) zeta potential of GNR-PEG-RGD shifted a little in positive direction due to slight positive charge of c(RGDfK). After modification with PEG and cRGD, the size of the nanostructure became bigger, so hydrodynamic diameter was increased. The shift in the zeta potential and hydrodynamic diameter indicated successful conjugation of PEG and c(RGDfK) peptides. UV-vis absorbance spectra showed no obvious change after PEG modification and cRGD conjugation with a maximum UV-vis absorption peak at around 780 nm (Figure 2(d)).

For radiolabeling of GNRs, the radiochemical yield of [^131^I]GNR-PEG-cRGD was 64.54 ± 3.81% (*n* = 4), and the radiochemical purity was 98.17 ± 0.86% (*n* = 4) after centrifugation. [^131^I]GNR-PEG-cRGD had favorable stability in vitro (Figure 2(e)) with radiochemical purities of 97.79 ± 0.50% in PBS and 95.59 ± 0.73% in FBS at 48 h after labeling.

### 3.2. Integrin *α*_v_*β*_3_ Expression and Cell Binding

**Figure 3:**
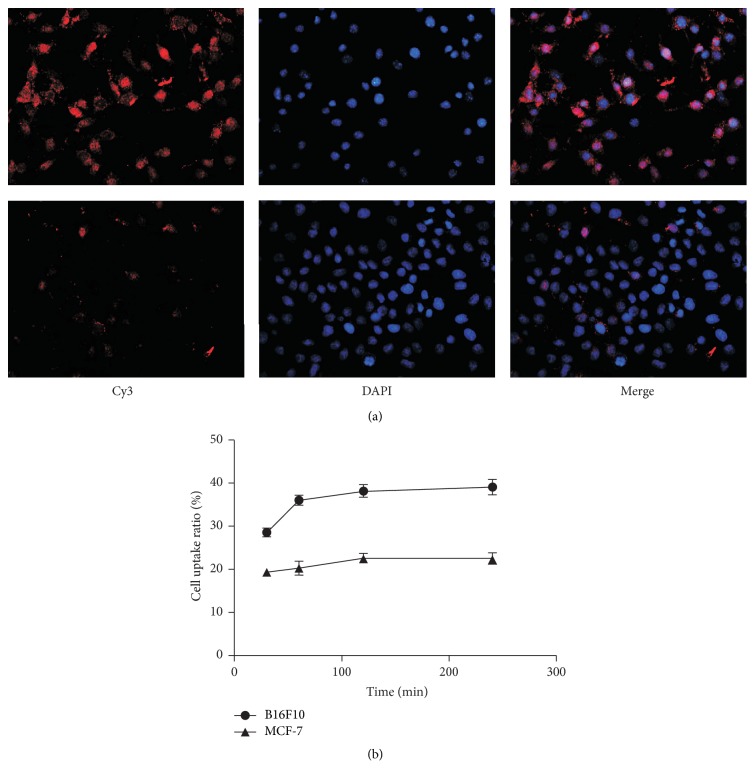
(a) Immunofluorescence staining of integrin *α*_v_*β*_3_ in B16F10 (upper row) and MCF-7 (lower row) cells. The nucleus were counterstained with DAPI. The red fluorescence intensity is proportional to the expression level of integrin *α*_v_*β*_3_ (×200). (b) Results of cell binding assays at various time points.

Immunofluorescence demonstrated that the integrin *α*_v_*β*_3_ expression level in B16F10 cells was significantly higher than that in MCF-7 cells (Figure 3(a)). Therefore, B16F10 cells were used as the experimental group, while MCF-7 cells were used as the negative control.

As shown in Figure 3(b), [^131^I]GNR-PEG-cRGD exhibited specific binding because the cell binging ratio of [^131^I]GNR-PEG-cRGD in B16F10 cells increased as time elapsed and reached a peak (38.20 ± 1.48%) at 120 min, while the accumulation of [^131^I]GNR-PEG-cRGD in MCF-7 cells was much lower than that in B16F10 cells (*p* < 0.05) with no obvious change over time. The specificity was also confirmed by receptor blocking experiments with an uptake ratio of 20.61 ± 1.15% at 120 min.

### 3.3. In Vivo Analyses

**Figure 4:**
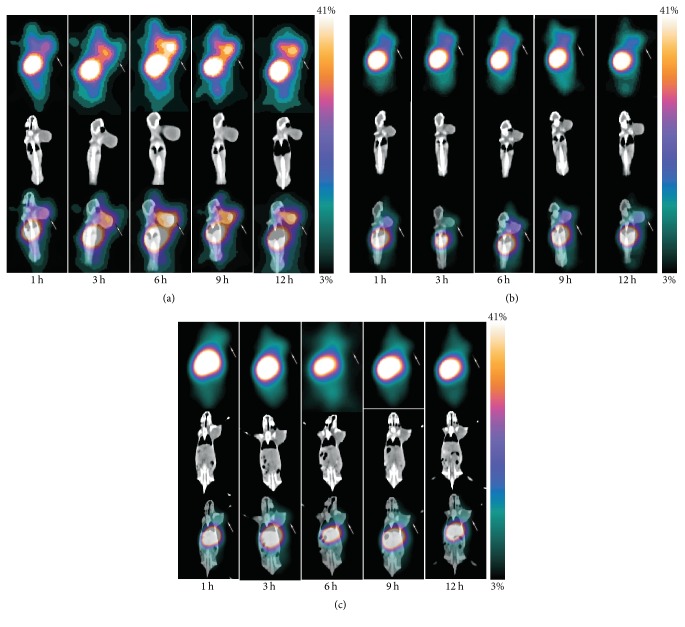
Representative whole body SPECT/CT images of B16F10 (a), blocked B16F10 (b), and MCF-7 (c) tumor-bearing mice at 1, 3, 6, 9, and 12 h after intravenous injection of [^131^I]GNR-PEG-cRGD. Arrows indicate tumor sites.

SPECT/CT images (Figure 4) and biodistribution analyses (Table 1) showed evident specific tumor uptake. [^131^I]GNR-PEG-cRGD had accumulated in B16F10 tumors quickly and effectively at 1 h after injection. Remarkably, the tumor uptake increased gradually over time and reached the peak value at about 6 h, and tumors were clearly visualized at 12 h after injection. However, nanoprobes in MCF-7 tumors were almost undetectable at all time points. In blocking experiments, B16F10 tumor uptake was clearly reduced. Biodistribution results revealed that B16F10 tumor uptake of [^131^I]GNR-PEG-cRGD was gradually increased to 5.09 ± 0.68 % ID/g (*n* = 4) at 6 h after injection, which was significantly higher compared with MCF-7 tumors (1.59 ± 0.39 % ID/g, *n* = 4, *p* < 0.05) and the blocked group (2.21 ± 0.52 % ID/g, *n* = 4, *p* < 0.05). Tumor/muscle ratios were 9.99 ± 2.98 (B16F10), 3.67 ± 0.92 (MCF-7), and 3.87 ± 0.93 (blocked) at 6 h. Liver, spleen, and lungs had remarkable radioactivity uptake. The kidneys showed low uptake of about 2 % ID/g.

### 3.4. Autoradiography and Immunofluorescence

**Figure 5:**
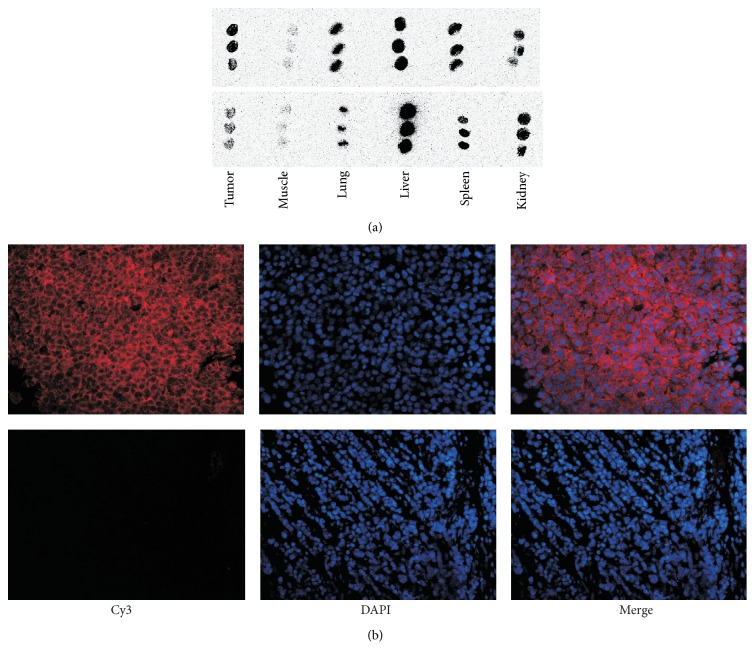
(a) Autoradiography of tumor tissue and organs. B16F10 tumor-bearing mice (upper row) and MCF-7 tumor-bearing mice (lower row). (b) Immunofluorescence staining of integrin *α*_v_*β*_3_ in B16F10 (upper row) and MCF-7 (lower row) tumors (×200).

Autoradiography also revealed abundant radioactivity accumulation in B16F10 tumors and little radioactivity accumulation in MCF-7 tumors (ROIs: 3.73 ± 0.75 versus 1.27 ± 0.47, *n* = 4, *p* < 0.05), which demonstrated specific targeting of the nanoprobe. High radioactivity had accumulated in samples of the lungs, liver, and spleen, while radioactivity distribution in muscles was sparse (Figure 5(a)). These ex vivo results were consistent with in vivo analyses.

Immunofluorescence staining of integrin *α*_v_*β*_3_ in B16F10 sections revealed intense fluorescence, but little fluorescence in MCF-7 sections (Figure 5(b)), which validated abundant integrin *α*_v_*β*_3_ expression in B16F10 tumors, but low expression in MCF-7 tumors.

## 4. Discussion

An ^131^I-labeled, cRGD-conjugated PEG-modified GNR probe was synthesized successfully. Compared with a reported spherical gold nanoprobe with single surface plasma resonance in visible light [12], which limits certain applications in the medical field, the probe we designed and synthesized possesses distinct advantages in the field of optical biological applications. Excellent surface plasma resonances in the NIR region confer GNRs with minimal light absorption by hemoglobin and water, maximal penetration [25], and highly efficient photothermal conversion [9], making them particularly attractive for optoacoustic imaging [13] and photothermal therapy [26].

There has been increasing use of radioisotopes to label nanoparticles, such as positron emitters copper-64 [26], gallium-67 [27], and zirconium-89 [28], and single photon emitters Technetiom-99m [3] and radioiodine (e.g., iodine-125 [29], iodine-123, and iodine-131 [30]). In most conditions, a bifunctional chelator, such as DOTA or HYNIC, must be conjugated to the nanoparticle for radiolabeling. Therefore, modification of nanoparticles is required before labeling. It has been shown that iodide ions have high affinity and strong binding to the surface of GNRs [8, 29, 31, 32]. Iodide ions absorb preferentially onto facets of GNRs and form the strongest bonds with Au, which probably leads to the formation of a surface of AuI [18]. Such simple chemistry between iodine and GNRs allows straightforward and efficient labeling of radioiodine to GNRs without iodination reagents or iodine-accepting functional groups such as a phenol residue [29]. Iodine-125 has been reported to directly label GNRs in a simple and rapid manner [8, 29]. Here, we first report the in vitro and in vivo behaviors of directly ^131^I-labeled GNRs by simply mixing a GNR colloid with [^131^I]NaI at room temperature for a short time. This reaction takes place fairly rapidly and completely, which agrees well with the previous reports of high affinity and strong binding of iodide ions to the surface of GNRs [18]. The labeling method is characterized by simplicity, a short reaction time, mild reaction conditions, and high yield. Another advantage is that purification is simple by centrifugation to remove free [^131^I]Iodide ions. Furthermore, this nanoprobe has excellent stability with radiochemistry purity greater than 95% after incubation in PBS or FBS for 48 h. ^131^I is widely used in clinics and easy to obtain. The properties of *β*-emitters are used for radiotherapy. Passing through tissue, the ejected *β*-particles (i.e., electrons) interact with atoms, mainly in water molecules, and lose their energy, leading to the generation of excited and ionized atoms and free radicals that are responsible for DNA damage in cells by inducing single-strand breaks in DNA [33], making ^131^I the optimal choice for application as a theranostic agent [19]. Our ultimate goal is to use this probe for both imaging and therapy, so ^131^I was chosen.

The significant difference in cellular uptake of [^131^I]GNR-PEG-cRGD by B16F10 and MCF-7 cells confirmed receptor-specific internalization of the probes, suggesting that the probes were taken up by tumor cells via receptor-mediated endocytosis. However, [^131^I]GNR-PEG-cRGD accumulation in MCF-7 cells and blocked B16F10 cells was up to about 20% because of passive uptake, which may be explained by the following three reasons. First, the nanometric size of the probe is known to be taken up by all mammalian cell types [34]. Second, serum proteins in the cell culture may be absorbed onto the surface of nanoparticles or targeted c(RGDfK) peptides, which can be taken up by cells and can mediate uptake of nanoparticles into cells nonspecifically via the mechanism of receptor-mediated endocytosis [34]. Third, tumor cell membranes have a predominant negative charge, whereas the zeta potential of [^131^I]GNR-PEG-cRGD was positive, which can bind efficiently to the surface of tumor cells by electrostatic attraction and then promote nonspecific uptake via clathrin-mediated endocytosis [35].

SPECT/CT imaging results revealed high [^131^I]GNR-PEG-cRGD accumulation in B16F10 tumors; the tumors of blocked B16F10 mice and MCF-7 mice are also visible although they are not very clear. In biodistribution study, tumor uptake values were 5.09 ± 0.68, 2.21 ± 0.52, and 1.59 ± 0.39 % ID/g at 6 h p.i. in B16F10, blocked B16F10 and MCF-7 mice, with tumor/muscle ratio of 9.99 ± 2.98, 3.87 ± 0.93, and 3.67 ± 0.92, respectively. These results indicate that low EPR effects existed in B16F10/MCF-7 bearing mouse due to the characteristics of nanoparticles and solid tumor, and tracer accumulation in B16F10 can be explained by both integrin *α*_v_*β*_3_-specific endocytosis and EPR effect, in which active targeting plays a vital role.

Theoretically, due to the large size of these nanoparticles, usually optimal uptake would be observed at late time points. Actually, our in vitro cell binding results were 28.52 ± 1.00%, 36.02 ± 1.20%, 38.2 ± 1.48%, and 39.11 ± 1.80% at 30, 60, 120, and 240 min and nearly reached a platform at 120 min; our in vivo imaging also revealed highest tumor uptake present at 6 h and decreased overtime. In addition, significant thyroid uptake is also observed which increased overtime in our studies; thyroid uptake was also observed in reported 125I-Labeled Gold Nanorods papers as expected [8, 29]. One possible explanation of short blood circulation (dropped at 6 h p.i.) and rapid thyroid accumulation (e.g., 1 h p.i.) was in vivo deiodination from nanoparticles. Our in vitro stability results may not reflect real condition, because iTLC might not be able to identify the disassociated[131I] that might also be absorbed by protein(s) in blood. We will use FPLC to further assess the serum and in vivo stability of [131I]GNR-PEG-cRGD, and late time points will be included in our therapy study.

Compared with other radiotherapeutic systems such as those based on Lu-177-gold nanoparticles-RGD, one study compared 177Lu-labeled monomeric, dimeric, and multimeric RGD peptides for the therapy of tumors expressing a(n)b(3) integrins; 177Lu-AuNP-c(RGDfK)C was demonstrated as the best one for targeted radionuclide therapy of tumors expressing a(n)b(3) integrins; with highest tumor uptake of 6.42 ± 0.71 % ID/g at 6 h [36], our [131I]GNR-PEG-cRGD system has similar in vivo stability (5.09 ± 0.68% ID/g at 6 h). Another study reports that the mean tumor residence times of 177Lu-AuNP-RGD were 61.6 ± 5.8 h [37]. And we will get the data in our therapy study.

Although CTAB was replaced with HS-PEG_(5000)_-COOH for better stability and biocompatibility, significant uptake was still observed in the liver and spleen because of abundant macrophages in the reticuloendothelial system of the liver and spleen and the colloidal nature of the probe, which have been well documented in previous reports of radiolabeled nanoparticles [3, 4]. Efforts have been made to understand and minimize uptake by the liver and spleen as much as possible. One study reported that targeting ligands on the surface of nanoparticles might even be detrimental because their exposure can accelerate nanoparticle opsonization and blood clearance by the immune system, resulting in high uptake in the liver and spleen [35]. Morales-Avila et al. [3] studied the biodistribution of GNPs using various administration methods. Their results showed that intravenous administration resulted in higher liver and spleen accumulation than intraperitoneal administration, because intravenous administration leads to opsonization followed by substantial uptake by macrophages located in the liver and spleen. Our results revealed that [^131^I]GNR-PEG-cRGD had accumulated in the liver and spleen at an early time point and gradually declined over time; the reason may be due to the radiolabeled nanosystem accumulation by reticuloendothelial system (RES), metabolized by the hepatobiliary system; it is also possible that the activity eliminated by the hepatobiliary system corresponds to the free iodide and not to the radiolabeled nanosystem.

However, the detailed metabolism mechanism in vivo is still not understood. The size of a nanoparticle may be another influencing factor. It has been reported that GNPs of less than 5-6 nm in size can be removed from the body via the kidney which can minimize nonspecific accumulation by RES [29]. In addition, the final metabolic pattern of larger sized nanoparticles is associated with the shape and surface chemistry [38]. Our results also demonstrated that the in vivo environment is far more complex than in vitro model systems.

In summary, a stable and tumor-specific SPECT imaging nanoparticle probe was successfully prepared in this study. The probe can specifically target integrin *α*_v_*β*_3_-expressing tumor cells both in vitro and in vivo mainly by receptor-mediated endocytosis. Importantly, the radiolabeling method is simple and fast with a high yield and high stability. These promising results demonstrate that our [^131^I]GNR-PEG-cRGD probe can be used as an angiogenesis-targeted SPECT imaging probe. Currently, more detailed studies to improve the in vivo fate of the [^131^I]GNR-PEG-cRGD probe and the use of this multifunctional probe as a theranostic agent are ongoing.

## 5. Conclusion

In this study, a smart nanoprobe, [^131^I]GNR-PEG-cRGD, was successfully developed, and it showed specific binding ability with integrin *α*_v_*β*_3_, indicating its potential as a multifunctional theranostic agent for tumors.

## Figures and Tables

**Table 1:** Biodistribution of [^131^I]GNR-PEG-cRGD in C57BL/6 mice with B16F10 tumors and athymic mice with MCF-7 tumors at 1, 3, and 6 h after intravenous administration (*n* = 4).

Tissue	B16F10			B16F10 blocking	MCF-7		
	1 h	3 h	6 h	6 h	1 h	3 h	6 h
Blood	3.53 ± 1.31	4.53 ± 1.58	3.19 ± 0.35	3.97 ± 1.25	3.14 ± 2.10	3.1 ± 0.31	2.44 ± 0.28
Brain	0.20 ± 0.09	0.18 ± 0.03	0.17 ± 0.02	0.15 ± 0.09	0.18 ± 0.09	0.15 ± 0.03	0.10 ± 0.02
Heart	1.14 ± 0.44	1.45 ± 0.27	0.85 ± 0.19	0.78 ± 0.17	0.45 ± 0.16	0.93 ± 0.17	0.47 ± 0.17
Lung	19.25 ± 4.59	11.74 ± 1.97	7.07 ± 0.25	6.93 ± 0.24	20.56 ± 9.24	17.41 ± 1.04	3.31 ± 1.87
Liver	32.05 ± 5.37	28.83 ± 7.17	26.51 ± 4.93	27.59 ± 5.01	30.18 ± 4.87	31.51 ± 5.71	27.60 ± 3.34
Spleen	11.28 ± 0.63	9.86 ± 1.05	5.55 ± 0.69	6.32 ± 1.01	15.99 ± 1.74	8.57 ± 0.70	6.20 ± 1.18
Kidney	2.34 ± 0.75	2.60 ± 1.04	2.29 ± 0.21	3.01 ± 0.91	2.29 ± 1.51	2.00 ± 0.36	1.50 ± 0.37
Stomach	3.59 ± 1.81	6.12 ± 2.28	3.43 ± 0.44	4.02 ± 0.59	3.49 ± 2.69	5.44 ± 2.75	4.70 ± 1.37
Intestine	1.52 ± 0.55	1.60 ± 0.57	1.23 ± 0.48	1.04 ± 0.29	1.15 ± 0.77	1.35 ± 0.25	0.89 ± 0.47
Muscle	0.47 ± 0.19	0.59 ± 0.08	0.51 ± 0.08	0.57 ± 0.12	0.54 ± 0.22	0.58 ± 0.23	0.42 ± 0.07
Bone	2.59 ± 0.78	3.29 ± 1.47	1.69 ± 0.13	1.70 ± 0.21	2.02 ± 0.85	2.72 ± 0.68	1.26 ± 0.71
Thyroid	2.88 ± 0.37	2.25 ± 1.09	2.93 ± 0.75	3.01 ± 0.92	3.26 ± 2.15	2.93 ± 0.26	3.59 ± 2.68
Tumor	3.57 ± 1.25	4.02 ± 1.45	5.09 ± 0.68	2.21 ± 0.52	1.31 ± 0.88	1.75 ± 0.26	1.59 ± 0.39
Uptake ratio							
Tumor/blood	1.01 ± 0.11	0.80 ± 0.29	1.58 ± 0.13	0.56 ± 0.22	0.52 ± 0.07	0.57 ± 0.04	0.63 ± 0.19
Tumor/muscle	7.06 ± 2.53	6.89 ± 2.37	9.99 ± 2.98	3.87 ± 0.93	2.17 ± 1.06	3.26 ± 0.98	3.67 ± 0.92
